# Investigating the Link Between Linguistic and Non-Linguistic Cognitive Control in Bilinguals Using Laplacian-Transformed Event Related Potentials

**DOI:** 10.1162/nol_a_00056

**Published:** 2021-12-23

**Authors:** Martha N. Mendoza, Henrike K. Blumenfeld, Robert T. Knight, Stephanie K. Ries

**Affiliations:** Helen Wills Neuroscience Institute and Department of Psychology, University of California, Berkeley, Berkeley, CA, USA; School of Speech, Language, and Hearing Sciences, San Diego State University, San Diego, CA, USA; Center for Clinical and Cognitive Neuroscience, San Diego State University, San Diego, CA, USA; SDSU-UCSD Joint Doctoral Program in Language and Communicative Disorders, San Diego, CA, USA

**Keywords:** bilingual language control, cognitive control, cross-linguistic interference, Laplacian transformation, event-related potentials

## Abstract

Bilinguals’ need to suppress the activation of their other language while speaking has been proposed to result in enhanced cognitive control abilities outside of language. Several studies therefore suggest shared cognitive control processes across linguistic and non-linguistic tasks. Here we investigate this potential overlap using scalp electroencephalographic recordings and the Laplacian transformation, providing an estimation of the current source density and enabling the separation of EEG components in space. Fourteen Spanish-English bilinguals performed a picture-word matching task contrasting incongruent trials using cross-linguistic false cognates (e.g., a picture – foot, overlaid with distractor text: the English word *PIE*, i.e., the false cognate for the Spanish *pie* meaning “foot”) with congruent trials (matching English picture names and words, i.e., a picture – foot, with overlaid text: the English word *FOOT*), and an unrelated control condition. In addition, participants performed an arrow-version of the Eriksen flanker task. Worse behavioral performance was observed in incongruent compared to congruent trials in both tasks. In the non-linguistic task, we replicated the previously observed congruency effect on a medial-frontal event-related potential (ERP) peaking around 50 ms before electromyography (EMG) onset. A similar ERP was present in the linguistic task, was sensitive to congruency, and peaked earlier, around 150 ms before EMG onset. In addition, another component was found in the linguistic task at a left lateralized anterior frontal site peaking around 200 ms before EMG onset, but was absent in the non-linguistic task. Our results suggest a partial overlap between linguistic and non-linguistic cognitive control processes and that linguistic conflict resolution may engage additional left anterior frontal control processes.

## INTRODUCTION

Investigating the cognitive consequences of bilingualism has become a source of great interest in the past few decades. Evidence from language perception and production tasks (e.g., lexical decision, picture naming) indicates that bilinguals’ languages are active in parallel (Colomé, 2001; Costa et al., 1998; Dijkstra & van Heuven, 1998; Green, 1998; Kroll et al., 2008; Marian et al., 2003; Marian & Spivey, 2003). In order to speak or understand an intended language, bilinguals must therefore be able to selectively inhibit or filter non-target language representations (for reviews, see La Heij, 2005; van Heuven & Dijkstra, 2010). The unique need bilinguals have to constantly regulate two languages has been proposed to result in enhanced cognitive control abilities that expand beyond the language domain (Bialystok et al., 2004, 2008; Bialystok & Craik, 2010; Kroll & Bialystok, 2013), and has been coined the *bilingual advantage* (BA). In support of this claim, a large number of studies have shown that bilinguals outperform monolinguals in non-linguistic tasks that make use of cognitive control processes (Bialystok et al., 2004, 2008; Costa et al., 2008, 2009). The underlying assumption is that these cognitive control processes are shared between language and other functions. Therefore, these cognitive control processes are adaptively “trained” by the constant need bilinguals have to suppress their other language or switch between their languages (Green & Abutalebi, 2013). However, a significant number of studies have also reported results in which bilinguals show no such advantage in the same non-linguistic tasks that have been used to show the BA (for reviews see Lehtonen et al., 2018; Paap & Greenberg, 2013). Therefore, the existence of these shared cognitive control processes in bilinguals has been questioned.

Brain imaging studies have sought to answer the question of whether there is a possible functional overlap between cognitive control and language tasks in bilinguals by investigating whether similar brain regions or electrophysiological components may be engaged in both types of tasks. However, determining when functional overlap across linguistic and non-linguistic tasks can be interpreted as constituting domain-generality in processing can be a matter of debate. Nozari and Novick (2017) argued that two criteria must be met for determining functional overlap and inferring domain-generality in processing: “*shared computational principles*” and “*shared neural implementation*.” Shared neural implementation can be captured, for example, through the overlap of brain activation across tasks in functional magnetic resonance imaging (fMRI) studies. Shared computational principles refer to similar processes operating in different domains on different domain-specific representations. As argued in Nozari and Novick (2017), it is undisputed that representations are domain-specific and are stored by different cortical regions. However, the processes operating upon these representations may be common across domains. In the comparison between non-linguistic and linguistic tasks, these processes are what is generally targeted. Even though shared neural implementation can be an argument in favor of shared computational principles, it may not always be sufficient. Additional information from imaging modalities beyond fMRI can help further support the existence of shared computational principles across domains. For example, the shared computational principles criterion may be supported by identifying event-related potential (ERP) components modulated in the same way across domains and emerging from the same general areas as those showing shared neural implementation. Below, we review fMRI and EEG studies investigating the functional overlap between linguistic and non-linguistic tasks in bilinguals, and discuss how the present study can bring further information in this respect.

FMRI studies in bilinguals have revealed functional overlap in terms of brain activation between cognitive control and language tasks (Coderre et al., 2016; Garbin et al., 2010; van Heuven & Dijkstra, 2010). Importantly, several brain imaging studies have found that bilinguals activate a brain network typically associated with executive control, including medial frontal regions such as the anterior cingulate gyrus (ACC), the pre-supplementary motor area (pre-SMA), left inferior prefrontal cortex, and the left caudate nucleus when they need to manage cross-linguistic conflict arising from the automatic lexical activation of the non-target language (Rodriguez-Fornells et al., 2005; van Heuven et al., 2008). Bilinguals have also been shown to activate brain regions typically associated with language tasks, such as the left inferior frontal gyrus, when performing non-linguistic cognitive control tasks (e.g., a non-linguistic switching task; Garbin et al., 2010). This cognitive control network underlying bilingual processing has been confirmed by large meta-analytic studies combining data on bilingual processing (Abutalebi & Green, 2007; Sulpizio et al., 2020). Overall, these studies suggest that similar brain regions are modulated by cognitive control demands in the linguistic and non-linguistic domains in bilinguals. In addition, the current literature linking language control during bilingual processing to a domain-general executive control network (for a review, see Calabria et al., 2018) aligns well with Fedorenko and Thompson-Schill’s (2014) view of a specialized “core” language system that is overlaid with a domain-general “peripheral” system.

In examining the overlap between linguistic and non-linguistic cognitive control networks in bilinguals, a central strategy is to identify language tasks where cross-linguistic interference has been documented during processing. False cognates, also known as interlingual homographs or homophones, provide a lens into bilingual processing contexts where cross-linguistic interference is present. For example, the English word for a baked treat, *pie*, means “foot” in Spanish, thus activating distinct semantic representations in each language of Spanish-English bilinguals. Behavioral evidence shows lower accuracy rates and longer reaction times in bilinguals for interlingual homograph processing relative to control words (e.g., van Heuven et al., 2008; Vanlangendonck et al., 2019; von Studnitz & Green, 2002). This finding has been interpreted as reflecting crosslinguistic interference at the lexico-semantic level as participants select a language-specific response. Using fMRI with a lexical decision task in a monolingual context, van Heuven et al. (2008) found enhanced blood-oxygen-level-dependent (BOLD) signal in areas of the executive control network, including the pre-SMA and ACC when Dutch-English bilinguals processed interlingual homographs (e.g., the word *room* is part of a house in English but means “cream” in Dutch; van Heuven et al., 2008). In a similar lexical decision study in Dutch-English bilinguals, Peeters et al. (2019) also identified activation in pre-SMA and left inferior frontal gyrus (IFG) associated with processing of interlingual homographs compared to English control words. However, showing a direct neural implementation overlap between linguistic and cognitive control networks requires using linguistic and non-linguistic tasks directly in the same participants. So far, only a few studies have used this direct comparison in the same participants (Coderre et al., 2016; De Baene et al., 2015; Ye & Zhou, 2009), and only Coderre et al. (2016) tested bilinguals specifically. In particular, overlapping activations in the arrow version of the Eriksen flanker task (Eriksen & Eriksen, 1974; Stoffels & van der Molen, 1988) and a semantic categorization task have been found in the left inferior frontal gyrus in bilinguals but not in monolinguals (Coderre et al., 2016), suggesting shared neural implementation across domains in bilinguals.

Although fMRI studies can inform us about whether or not similar brain regions are engaged in linguistic and non-linguistic control, studying the time point at which this functional overlap takes place in the bilingual brain requires temporally resolved techniques, which could help identify shared computational principles. Previous studies using EEG and, in particular, ERPs have indirectly and directly investigated this issue using a variety of paradigms, including language switching paradigms (e.g., G. M. Jackson et al., 2001), go/no-go tasks manipulating interference at different levels (Rodriguez-Fornells et al., 2006), and the negative priming paradigm (Dash & Kar, 2020). These studies have often reported fronto-central activity peaking between 200 and 500 ms post-stimulus onset (N2 and N400 components) as being larger in situations requiring more control (e.g., switch trials, no-go trials, cross-linguistic incongruent trials) compared to situations requiring less control (e.g., non-switch trials, go trials, cross-linguistic congruent trials) in linguistic and non-linguistic tasks (S. R. Jackson et al., 1999). However, similarly as for fMRI studies, few studies have directly compared ERPs associated with cognitive control processes in linguistic and non-linguistic tasks in the same participants. A recent study (Dash & Kar, 2020) used the negative priming paradigm and an animacy judgment task, with words and pictures in the linguistic versus non-linguistic versions respectively. The results showed modulations of the N2 amplitude in the linguistic and non-linguistic versions alike, suggesting a computational (processing) overlap between linguistic and non-linguistic domains in bilinguals. While these results are informative, the non-linguistic task used pictures representing nameable items, which may not have been ideal given the possible internal word retrieval process induced by the stimuli. Another possible more general limitation stems from the limited spatial resolution of traditional ERP studies, making it difficult to observe components that could be differentially affected by linguistic compared to non-linguistic interference at similar time points.

Previous non-linguistic electrophysiological studies using the Laplacian transformation have described ERPs associated with cognitive control mechanisms time-locked to the response. The Laplacian transformation provides an estimate of the current source density and therefore more focal topographic resolution than traditional ERPs (Babiloni et al., 2001). Using this technique has enabled the dissociation of activity occurring at neighboring sites but attributable to different cognitive processes. One of these components, the N-40, is of particular interest here. The N-40 is a negative going wave that peaks around 40 ms prior to electromyographic (EMG) onset leading to the response (i.e., the button press), maximal over medial-frontal electrodes (FCz), and preceding the components associated with response execution and inhibition recorded over the contralateral and ipsilateral motor cortices respectively (Vidal et al., 2003). Critically, these later motor components cannot be dissociated from the N-40 in traditional monopolar ERP analyses, which may lead to a confound between these activities if Laplacian transformation is not used (for a demonstration, see Burle et al., 2015). By using Laplacian transformation and recording the EMG activity associated with the response, this component was found to be more closely aligned in time to the onset of the EMG activity leading to the response than to the button press itself (Vidal et al., 2003). The N-40 is larger in incongruent than congruent trials in the Eriksen flanker task (Roger, 2009). The N-40, along with the inhibition of the ipsilateral motor cortex associated with the hand not being used, also disappears when there is no choice to be made between two possible responses (in go/no-go tasks; Vidal et al., 2011). In addition, the amplitude of this component is reduced when biasing information about the response to be produced is available to the participant (Carbonnell et al., 2004). The N-40 has therefore been associated with decision-making processes, and in particular response selection, in choice reaction time tasks (Vidal et al. 2011), where participants must decide between two conflicting responses, usually a left or right button press. Importantly, the N-40 is dissociable from the lateralized readiness potential, which is lateralized and peaks around response onset (for a review, see Vidal et al., 2018). Moreover, as mentioned earlier, the N-40 peaks around 40 ms prior to EMG onset leading to the response (i.e., the button press), and hence is also different from the error-related negativity (ERN), which peaks after the response, and which has been associated with action monitoring, including language output monitoring (e.g., Riès et al., 2011, 2020; Riès, Xie, et al., 2013) and conflict monitoring (e.g., Masaki et al., 2012; Yeung et al., 2004). Although Laplacian transformation does not allow the identification of specific neural sources, the fronto-central distribution of the N-40 is compatible with the engagement of medial frontal regions in cognitive control (for a review, see Ridderinkhof et al., 2011).

In this study, we used the same Laplacian transformation technique and compared a linguistic decision-making task, indexing cross-linguistic interference using interlingual homographs, to a non-linguistic decision-making task. In the current study, we will refer to interlingual homographs as false cognates to focus on the competing meanings that are activated by these words across languages. We hypothesize that if bilinguals use shared cognitive control processes in the language domain and outside of language, then this overlap may be visible on the N-40 component. More specifically, we hypothesize that a negative component will be observed in our linguistic decision-making task at the same fronto-central recording site as in the non-linguistic decision-making task and that this component will be sensitive to the congruency manipulations in both tasks. Finding a similar fronto-central decision-making mechanism engaged in linguistic and non-linguistic domains would provide support for the idea that similar cognitive control processes are involved in language and outside of language, providing a further argument in favor of shared neural computations in language and outside of language in bilinguals. We have previously used Laplacian transformation to examine the brain dynamics underlying picture naming and found two main frontal components, one fronto-central peaking between 300 and 200 ms before vocal onset, as well as a left frontal component peaking around vocal onset (Riès, Janssen, et al., 2013). While this previous study was mainly descriptive, did not target bilinguals, and did not include a non-linguistic task, we are expecting to find similar medial frontal and left frontal components in the linguistic task in the present study. Whether or not these components will be similarly sensitive to non-linguistic and linguistic manipulations will be tested here.

### The Current Study

We recorded scalp EEG in 17 Spanish-English participants as they performed a picture-word matching (PWM) task, and the arrow version of the Eriksen flanker task (Eriksen & Eriksen, 1974; Stoffels & van der Molen, 1988). In the PWM task, participants decided whether a picture, and a word superimposed on it, corresponded to the same word or not. The PWM contrasted incongruent trials using false cognates (e.g., picture – foot; distractor: English word *PIE*, i.e., false cognate for the Spanish *pie* meaning “foot”) with congruent trials (matching picture name and word) and an unrelated control condition. We also employed the arrow version of the flanker task, where participants decided on the direction of a central arrow while ignoring flanking arrows. In incongruent trials, flanking arrows were in the opposite direction of the central arrow, while they matched in congruent trials. We preferred the arrow version of the Eriksen flanker task over the original version (using letter stimuli) as we aimed to make this task as non-linguistic as possible.

We aligned the linguistic PWM task with the non-linguistic Eriksen flanker task in terms of their underlying loci of cognitive control. Specifically, the incongruent conditions on both tasks were characterized as including stimulus-level and response-level conflict, sources of conflict that are considered separable during bilingual processing (Dijkstra & van Heuven, 2002) and in non-linguistic cognitive control tasks (Kornblum et al., 1999). Stimulus- and response-based conflict was specifically examined in false cognates by van Heuven et al. (2008): Stimulus-based conflict (i.e., the two possible interpretations of false cognates across languages) was associated with activity in anterior and posterior left inferior prefrontal cortex; response-based conflict (i.e., the conflict generated when participants had to explicitly identify language membership of the false cognates at the response level) was associated with activity in pre-SMA and ACC. Similarly, the incongruent condition of our flanker task contains stimulus-based conflict (i.e., the presence of right-pointing and left-pointing arrows around a central arrow on the display) and response-level conflict (e.g., the mapping of a right-hand response, despite the presence of left-pointing arrows on the display).

In both the PWM and flanker tasks, participants pressed buttons using their thumbs to give their answers, and EMG activity of the corresponding muscles (*flexor pollicis brevis*) was recorded along with scalp EEG (as in, e.g., Riès et al., 2011; Roger, 2009; Vidal et al., 2011). EMG recordings were used to mark the onset of the muscular activity associated with pressing the response buttons. Laplacian-transformed ERPs were time-locked to EMG onset to investigate the potential overlap between medial frontal cognitive control processes in linguistic and non-linguistic decision-making.

We focused on the medial frontal and left frontal components previously described in linguistic (Riès, Janssen, et al., 2013) and non-linguistic tasks (Vidal et al., 2011). In particular, we focused on the N-40 component as this component has been previously described in non-linguistic tasks focusing on cognitive control, and its amplitude has been shown to be modulated by congruency in the flanker task (Roger, 2009). We hypothesized that a larger N-40 would be observed in incongruent than in congruent trials in the flanker task as in Roger (2009). If the same underlying mechanism is similarly engaged in linguistic decision-making and is sensitive to cross-linguistic interference, then the N-40 should also be larger in the false-cognate compared to the congruent (i.e., matching) condition in the PWM task.

We further investigated whether additional left frontal activity would be sensitive to congruency in the PWM task and whether this left frontal activity would be selectively engaged in the linguistic task. Based on the results from the bilingual cognitive control literature (Abutalebi & Green, 2007; Sulpizio et al., 2020), we expected to find a left-lateralized ERP component sensitive to congruency in both the linguistic and non-linguistic tasks and reflecting the overlap in cognitive control function subserved by the left inferior frontal cortex. Left frontal activity has been previously reported in picture naming using the Laplacian transformation (Riès, Janssen, et al., 2013); therefore we expected to find similar activity in the present study in the linguistic task. Whether or not this same component is also present in non-linguistic tasks and is sensitive to cognitive control demands in linguistic and non-linguistic tasks remains to be investigated.

## METHODS

### Participants

The study was performed in agreement with the Declaration of Helsinki. All participants gave informed consent approved by the University of California, Berkeley, Committee for Protection of Human Subjects before the experiment.

A total of 17 right-handed Spanish-English bilinguals with normal or corrected to normal vision participated in the experiment (mean age = 20.41 years, *SD* = 1.12 years). The data of 3 participants were not included in the analysis because of failing to complete the experiment (*n* = 1) or technical difficulties during EEG recording causing the EEG data to be unusable (*n* = 1) or absent in one of the tasks (*n* = 1). Hence, the data of 14 participants (4 males, mean age = 20.21 years, *SD* = 1.05 years) was processed and analyzed for the current study. This number of participants is in line with previous studies of decision-making and response selection using linguistic and non-linguistic tasks describing similar components using EEG and Laplacian transformation (Carbonnell et al., 2004; Riès, Janssen, et al., 2013; Vidal et al., 2003, 2011).

All participants were students at the University of California, Berkeley. They were recruited through the Research Participatory Program (RPP) from the psychology department and received course credit or monetary compensation for their participation. The recruitment process was conducted in English, and participants were recruited specifically because they were Spanish-English bilinguals (Spanish was their first/native language). More specifically, the recruitment language indicated that participants must be at least 18 years of age, be a Spanish-English bilingual (specifying that Spanish must be their first language), be fluent in English and Spanish and use both languages at similar levels, be right-handed, and have normal or corrected-to-normal vision. All of the participants reported learning Spanish as their first language and English as their second language. The mean age for acquisition of English was 5.89 years of age (*SD* = 3.37). They all self-reported being more dominant in English at the time of testing, although speaking Spanish regularly. All interactions, questions, and instructions were kept in English throughout the participants’ involvement in the experiment.

### Materials and Design

#### Flanker task

Stimuli consisted of five arrows presented in white on a black background at the center of the screen, presented in free viewing within a visual angle of 7°. There were four stimulus conditions: congruent right (>>>>>), congruent left (<<<<<), incongruent right (<<><<), and incongruent left (>><>>). In analyses, congruent trials were compared to incongruent trials, with each condition including right- and left-facing arrow trials. There was a total of eight blocks containing 60 trials each, and a total of 120 trials per condition (i.e., congruent left, congruent right, incongruent left, and incongruent right) overall, hence the ratio of congruent to incongruent trials was 1 to 1 in this task. Stimuli were pseudorandomized, such that identical trials were not repeated more than 5 times in a row.

#### Picture-word matching task

Thirty-two colored pictures of common objects were selected as stimuli from the Bank of Standardized Stimuli (BOSS; Brodeur et al., 2010). All stimuli were presented at the center of a computer monitor.

**Figure 1.**
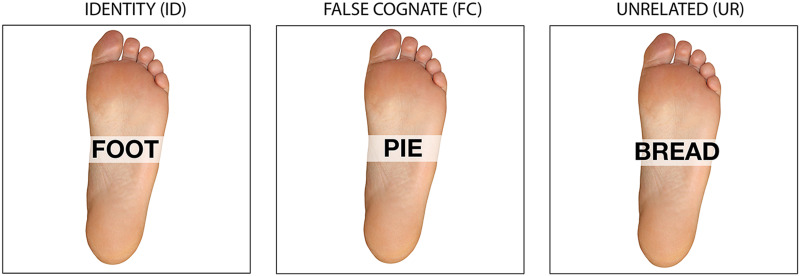
Picture-word matching task example stimuli in the Identity (ID) condition (the picture name and the distractor word match), the False Cognate (FC) condition (the picture name and the distractor word do not match and are false cognates, *pie* means “foot” in Spanish but not in English), and the Unrelated (UR) condition (the picture name and the distractor word do not match and are unrelated phonologically and semantically). In the FC condition, while participants might be tempted to respond that the picture and overlaid word are matches since the English word *PIE* is a perfect homograph with the Spanish translation equivalent of *foot*, this response would be incorrect.

Stimuli consisted of color pictures of common items fitted to a 2,000 × 2,000 pixel, white square background, superimposed with words centered to the center of the square, written in black bolded Arial font, 180 pixels high, over a transparent white rectangle (opacity: 65%), and were presented in free viewing within a visual angle of 7°. There were three stimulus conditions: a Congruent or Identity (ID) condition, in which the picture and the word matched (i.e., picture – foot; word – *foot*); an Incongruent or False-Cognate (FC) condition, where the picture and the word did not match in the target language, English, but where the word was a false-cognate to the Spanish picture-name (i.e., picture – foot; word – *pie*; Spanish picture-name = *pie* meaning “foot”; false cognate: English *pie* and Spanish *pie*); and an Unrelated control (UR) condition, where the picture and the word did not match in either language (i.e., picture – foot; word – *bread*) (see Figure 1). Thus in the FC condition, while bilingual participants might have been tempted to respond that the picture and overlaid word were matches since the English word *PIE* was also the Spanish translation equivalent of *foot*, this response would have been incorrect. Across the stimulus set of 32 target pictures with their corresponding false cognate distractor words, three false cognates were identical homographs of the picture’s Spanish translation equivalent (*bread* – *PAN*; *foot* – *PIE*; *net* – *RED*). (See the Appendix, located in the Supporting Information at https://doi.org/10.1162/nol_a_00056.) The remaining false cognates were near-homographs of the picture’s Spanish translation equivalent (e.g., *candle* [Spanish *vela*] – *VEIL*; *elbow* [Spanish *codo*] – *CODE*). There was a total of four blocks with 96 trials each. Each stimulus of the 32 unique stimuli was repeated 3 times per block, once in each condition (ID, FC, UR); hence, the rate of match (“yes” responses on ID trials) compared to no-match responses (“no” responses on FC and UR trials) was 1 to 2 in this task. Stimuli were pseudorandomized within blocks such that 2 consecutive stimuli did not share the same phonological onset and were not semantically related.

### Procedure

#### Behavioral recordings

Reaction times and accuracy were recorded using E-Prime’s Serial Response Box (SRB). Participants were first familiarized with the stimuli and the conditions in each task. The E-prime 2.0 Professional software (Psychology SoftwareTools, Inc., Pittsburg, PA) was used to control stimulus presentation for both tasks. Participants were comfortably seated at eye level to the computer monitor on which the stimuli were presented in a soundproof, dimly lit room. All participants completed the flanker task and the PWM task. Half of the participants performed the flanker task first. Participants were instructed to respond as fast and as accurately as possible. They were also told that the target language for the PWM task was English, and all interactions between the experimenters and participants were in English. Furthermore, participants were instructed to remain as still as possible during experimental blocks in order to prevent electromyographic (EMG) activity from contaminating the EEG signal as much as possible. Participants were able to rest for as long as needed between blocks in each task.

##### Flanker task.

A trial consisted in the following: (1) a fixation cross (a picture of a white plus sign on a black background) was displayed for 1,000 ms at the center of the screen; (2) the stimulus was presented on a black background for 120 ms; (3) a blank black screen displayed for 1,000 ms after stimulus offset. Participants were instructed to indicate the direction of the central target arrow by pressing a left or right button on the response box (the buttons were marked “left” and “right”). Responses could be made during stimulus presentation or during the blank screen, but the stimulus and blank screen remained even after the response had been made in order to maintain coherent trial structure.

##### Picture-word matching task.

A trial consisted in the following: (1) a fixation cross (a picture of a black plus sign on a white background) was presented for 1,000 ms at the center of the screen; (2) the stimulus was presented for 1,000 ms; (3) a blank screen was presented for 2,000 ms after the stimulus offset. Participants were instructed to indicate whether the picture and the word matched by pressing a right or left button marked “Match” or “No Match” on the corresponding SRB keys. Similar to the flanker task, responses could be made during stimulus presentation or during the blank screen.

#### Electrophysiological recordings

Electroencephalography was recorded using 64 Ag/AgCI pre-amplified electrodes (BIOSEMI, Amsterdam, Netherlands; 10–20 system positions). The sampling rate was 1024 Hz (with acquisition filters: DC to 208 Hz, 3 db/octave). Two surface electrodes (Ag/AgCl) were placed around 2 cm apart on the skin of the thenar eminence to record the EMG activity from the thumb muscle, flexor pollicis brevis. The vertical electrooculogram (EOG) was recorded by two surface electrodes (Ag/AgCl) placed above and below the left eye. Horizontal EOG was recorded by two electrodes placed next to the outer canthi. The passive reference electrode was placed over the right mastoid.

### Data Preprocessing

#### Behavioral data

Trials were considered errors when participants pressed the wrong button, did not produce a response, or when the produced response occurred later than 1,120 ms for the flanker task or 3,000 ms for the PWM task, respectively, corresponding to the time the stimulus and following white screen were presented in each task. Importantly, these cutoffs were well over 3 standard deviations above the mean reaction time (RT) in each task (Mean RT + 3 * *SD* in flanker = 701.5 ms; Mean RT + 3 * *SD* in PWM = 1,284.6 ms). Reaction times were measured between the time of stimulus presentation and the time of the button press.

#### EEG and EMG data

After data acquisition, EMG data were filtered (high pass = 10 Hz; low-pass = 300 Hz), and rectified. EMG onsets were manually marked on a trial-by-trial basis. Importantly, EMG onsets were clearly discriminable from the baseline. Trials containing more than one EMG burst before the button press were rejected from further analyses.

The EEG data were resampled to 256 Hz and vertical eye movements (i.e., eye blinks) were removed using independent component analysis as implemented in EEGLAB (Delorme & Makeig, 2004). A blind source separation algorithm based on canonical correlation analysis (BSS-CCA; De Clercq et al., 2006; de Vos et al., 2010) was applied on non-overlapping consecutive 30-sec time windows on monopolar recordings in order to remove any EMG activity (due to frowning or other muscular tension) that may have contaminated the EEG data.

Following the BSS-CCA procedure, the data were carefully inspected on a trial-by-trial basis, and all other artifacts were manually removed on monopolar recordings. Monopolar recordings for correct trials were then averaged to EMG onset, and a Laplacian transformation (approximating the current source density) was implemented in BrainVision Analyzer (Brain Products™, Munich) and applied to each participant's average (as in Riès et al., 2011, 2015, 2020; Riès, Janssen, et al., 2013; Riès, Xie, et al., 2013). The main advantage of using Laplacian transformation is that it is reference-free, and it improves the spatial definition as it enhances the separation of EEG components on the scalp, providing a good estimation of the corticogram (Nunez & Srinivasan, 2006). The baseline for EMG-locked averages was taken from 500 ms until 300 ms before EMG onset although the measures we used for analyses were independent from the baseline choice.

### Analysis

Statistical analyses were performed within R version 3.6.3 (R Core Team, 2020).

#### Behavioral data

We used the package lme4 to compute generalized linear (for reaction times) and logistic (for accuracy rates) mixed-effects models (Baayen et al., 2008; Jaeger, 2008), which rely on single-trial data rather than on averages over participants or items, and are also free from the assumptions of homogenous variance and sphericity that are inherent to the more classic ANOVA (Pinheiro & Bates, 2000). The individual reaction times were inversed to reduce skewness and approach a normal distribution. The analyses were performed on inversed reaction times and accuracy. We tested for a fixed effect of Condition (Congruent vs. Incongruent in the flanker task, and Identity vs. False Cognate vs. Unrelated in the PWM task) and controlled for random effects of picture name and participant, as well as by-item and by-participant random slopes for Condition. The *p* values were obtained using type-II analyses of deviance tables providing Wald chi-square (Wald χ^2^) tests and associated *p* values for the fixed effects in the generalized linear mixed-effects models, using the R package car (Fox & Weisberg, 2011). For all models, we report Wald χ^2^ values and *p* values from the analysis of deviance tables as well as raw beta estimates (β_raw_), 95% confidence intervals around these beta estimates, standard errors, *t* values for reaction times, Wald *z* values for accuracy rates, and associated *p* values.

Finally, we calculated Spearman correlation coefficients to examine a possible relation between congruency effects on flanker and PWM tasks on average reaction times and on error rates.

#### EEG data

We focused our analysis on the Laplacian-transformed EEG components time-locked to EMG onset on two sets of recoding sites: (1) the medial-frontal electrode, FCz, previously associated with cognitive control mechanisms in non-linguistic tasks (Vidal et al., 2011), and (2) the left frontal electrodes including F3, F7, AF3, and AF7, given activity seen on the grand averages at these recording sites. Even though our previous study found activity at FC5 (Riès, Janssen, et al., 2013), no activity was visible at this recording site on the grand averages in the present study. In order to assess the presence of an activity at these recording sites, we first compared the slope of the waveforms to zero (as in Riès et al., 2011, 2020; Riès, Janssen, et al., 2013; Riès, Xie, et al., 2013). Then, in order to assess whether any effects were present on the signal recorded at these electrodes, we calculated the peak-to-peak amplitudes and the latencies of the peaks of interest of the observed activities. These measures are known to be independent from the baseline. This analysis was performed on activities present within 500 ms prior to EMG onset. Peak latencies and peak-to-peak amplitude measures were defined as follows: The latencies of the peaks were measured on smoothed data, using a 40-ms-long sliding smoothing window to minimize the impact of background noise, on the grand average activities in the False Cognate and Incongruent conditions respectively as these conditions yielded the largest components on the grand averages. The surfaces between the *x* axis and the peaks of interest were calculated on 40-ms time windows around these latencies on the non-smoothed data for all conditions per participant. Finally, the peak-to-peak amplitude was calculated by finding the difference between the positive and negative surfaces (as in Riès et al., 2011, 2020; Riès, Janssen, et al., 2013; Riès, Xie, et al., 2013). These measures were calculated for electrodes F3 (the only left frontal electrode showing significant activity on the slope analyses) and FCz in the PWM and flanker tasks. All measures were compared using two-tailed paired Student’s *t* tests.

## RESULTS

We present the results for the flanker task first given our aim to compare cognitive control processes supporting language to cognitive control processes engaged outside of language.

### Behavioral Results

#### Flanker task

The mean RTs, accuracy rates, and standard deviations are presented per condition in Table 1A. There was a significant effect of condition on reaction times (Wald χ^2^(1) = 141.35, *p* < 0.001). Reaction times were longer in the Incongruent condition than in the Congruent condition (β_raw_ = −2.13 × 10^−4^, 95% CI [−2.48 × 10^−4^, −1.78 × 10^−4^], *SE* = 1.79 × 10^−5^, *t* = −11.89, *p* < 0.001). There was also a significant effect of condition on accuracy (Wald χ^2^(2) = 36.25, *p* < 0.001). The accuracy rate was lower in the Incongruent condition than in the Congruent condition (β_raw_ = −1.45, 95% CI [−1.92, −0.98], *SE* = 0.241, *z* = −6.02, *p* < 0.001).

**Table 1.** Mean reaction time and accuracy rate per condition and per task with standard deviations around the means in parenthesis.

**A. Flanker task**		
	Mean reaction time	Mean accuracy rate
Congruent	394 ms (*SD* = 55 ms)	99% (*SD* = 2%)
Incongruent	466 ms (*SD* = 51 ms)	82% (*SD* = 16%)

**B. Picture-word matching task**		
	Mean reaction time	Mean accuracy rate
Identity	715 ms (*SD* = 104 ms)	94% (*SD* = 8%)
False-cognate	747 ms (*SD* = 97 ms)	94% (*SD* = 7%)
Unrelated	714 ms (*SD* = 84 ms)	98% (*SD* = 3%)

#### Picture-word matching task

The mean RTs, accuracy rates, and standard deviations are presented per condition in Table 1B. There was a significant effect of condition on reaction times (Wald χ^2^(2) = 15.13, *p* < 0.001). Reaction times were longer in the False Cognate condition than in the Identity condition (β_raw_ = −1.58 × 10^−5^, 95% CI [−4.10 × 10^−5^, 9.42 × 10^−6^], *SE* = 1.29 × 10^−5^, *t* = −1.23), and longer in the False Cognate condition than in the Unrelated condition (β_raw_ = −1.75 × 10^−5^, 95% CI [−3.25 × 10^−5^, −2.53 × 10^−6^], *SE* = 7.64 × 10^−6^, *t* = −2.29). There was also a significant effect of condition on accuracy rates (Wald χ^2^(2) = 14.32, *p* < 0.001). The accuracy was lower in the False Cognate condition than in the Unrelated condition (β_raw_ = −0.86, 95% CI [−1.31, −0.41], *SE* = 0.23, *z* = −3.76, *p* < 0.001), and higher in the False Cognate than in the Identity condition (β_raw_ = 0.58, 95% CI = [0.11, 1.05], *SE* = 0.24, *z* = 2.44, *p* = 0.015). We note, however, that the median error rates were smaller than 5% across conditions (see Table 1).

#### Flanker versus PWM congruency effect comparison

There was no significant correlation between the size of the congruency effect in the flanker (Incongruent − Congruent) versus the PWM (False Cognate − Identity) tasks on reaction times (rho = −0.14, *S* = 518, *p* = 0.638) or accuracy rates (rho = −0.41, *S* = 643, *p* = 0.142). In addition, the random slopes of the corresponding condition effects per participant extracted from the mixed effect models were not correlated (RTs: rho = −0.068, *S* = 486, *p* = 0.820; accuracy rates: rho = −0.138, *S* = 518, *p* = 0.638).

### Electrophysiological Results

#### EEG results in the flanker task

**Figure 2.**
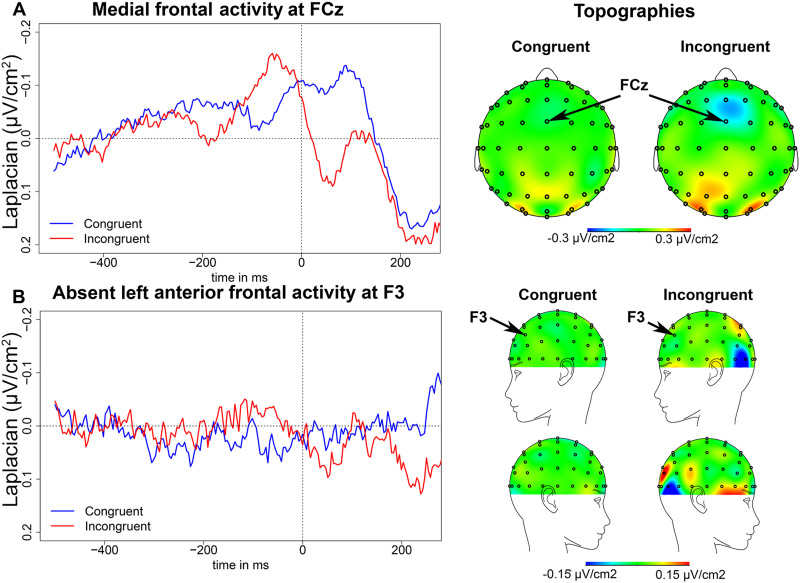
Flanker task EEG results. (A) Medial frontal activity in the flanker task. On the left, waveforms of the medial frontal activity at FCz in the Congruent (blue) and Incongruent (red) conditions time-locked to EMG onset (in ms). On the right, topographies of the medial frontal activity in each condition on a 50-ms time window centered around the peak latency in the Incongruent condition. (B) Absence of the left anterior frontal activity at F3; waveforms are shown on the left and on the right are shown the topographies on a 50-ms time window centered around the peak latency in the False Cognate condition in the picture-word matching task.

We observed a negativity at the fronto-central recording site, FCz, peaking on average 52 ms (*SD* = 32 ms) before EMG onset in incongruent trials (Figure 2A). The slope of this negativity was significantly different from zero between 150 and 50 ms before EMG onset in incongruent trials (*t*(13) = − 2.51, *p* = 0.026), replicating the N-40 component described in prior studies (e.g., Vidal et al., 2011). The peak-to-peak amplitude of this negativity was significantly larger in incongruent than in congruent trials (*t*(13) = 3.58, *p* = 0.003, with measures taken around the peak latencies in the Incongruent condition). Interestingly, in congruent trials, there was no negative component peaking around 40 ms prior to EMG onset. Indeed, the slope was not different from zero between 150 and 50 ms before EMG onset in congruent trials (*t*(13) = 1.07, *p* = 0.305), indicating that the N-40 component was sensitive to congruency manipulation.

There was a later negativity in congruent trials peaking on average 4 ms (*SD* = 32 ms) before EMG onset (the slope of the negativity was significantly different from zero between 100 before EMG onset and EMG onset, *t*(13) = −2.92, *p* = 0.012). However, the peak latency of this later negativity appears incompatible with the N-40 given that the N-40 typically peaks earlier (i.e., around 40 ms before EMG onset; e.g., Vidal et al., 2003). Of note, this later negativity was smaller in amplitude than the negativity peaking around 50 ms before EMG onset in incongruent trials (*t*(13) = 2.44, *p* = 0.030).

Following EMG onset, a negativity peaking around 100 ms post-EMG onset is visible in both conditions, which likely corresponds to the ERN previously shown to be present in correct trials as well as in incorrect trials, only of larger amplitude in incorrect trials (Vidal et al., 2000, 2003; Roger et al., 2010). However, the ERN was not the focus of the present study, hence no analyses were performed for this component.

The topography in Figure 2 indicates the fronto-central activity may also be present at Fz, anterior to FCz. We therefore also analyzed the activity recorded at Fz (see Figure S1) and found that the slope of the negativity at Fz between 150 and 50 ms before EMG onset was significantly different from zero in the Incongruent condition (*t*(13) = −4.62, *p* < 0.001), but not in the Congruent condition (*t*(13) = −0.96, *p* = 0.353). There was a significant difference between the slope of the negativity in incongruent versus congruent trials (*t*(13) = −3.89, *p* = 0.002; it was more negative-going in the Incongruent compared to the Congruent condition), but there was no significant difference between conditions for the peak-to-peak amplitude (*t*(13) = 2.05, *p* = 0.062).

There was no left anterior component at electrode F3 in the flanker task (Figure 2B). The slope of the waveforms was not significantly different from zero between 300 and 200 ms before EMG onset in the Congruent or Incongruent conditions (*t*(13) < 1). There was also no amplitude difference between the two waveforms (*t*(13) < 1; surface measures were taken between 200 and 100 ms before EMG onset).

#### EEG results in the PWM task

As in the flanker task, we observed negativities at the fronto-central recording site, FCz, peaking around 150 ms before EMG onset in the PWM task. For a parallel comparison with the flanker task, we first compare the False Cognate to the Identity condition and then describe the results of the Unrelated condition separately.

**Figure 3.**
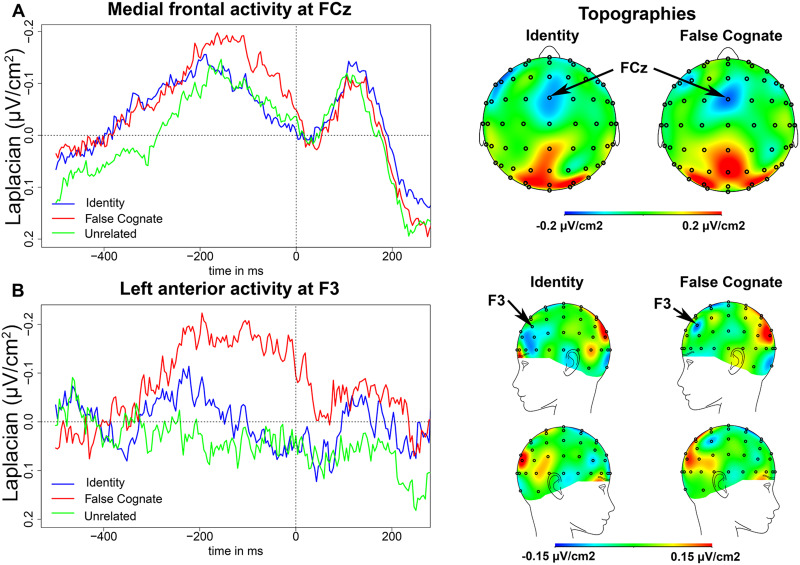
Picture-word matching task EEG results. (A) Medial frontal activity in the picture-word matching task. On the left, waveforms of the medial frontal activity at FCz in the Identity (blue), False Cognate (red), and Unrelated (green) conditions time-locked to EMG onset (in ms). On the right, topographies of the medial frontal activity in the Identity and False Cognate conditions on a 50-ms time window centered around the peak latency in each condition. (B) Left anterior frontal activity in the picture-word matching task. On the left, waveforms of the left anterior frontal activity at F3 in the Identity (blue), False Cognate (red), and Unrelated (green) conditions time-locked to EMG onset (in ms). On the right, topographies of the left anterior frontal activity in the Identity and False Cognate conditions on a 50-ms time window centered around the peak latency in each condition.

The negativity peaked on average 191 ms (*SD* = 79 ms) before EMG onset in the Identity condition, and 137 ms (*SD* = 69 ms) before EMG onset in the False Cognate condition (Figure 3A). There was an effect of condition on the latency of the peak (*t*(13) = 2.53, *p* = 0.025), as the negativity peaked later in the False Cognate condition than in the Identity condition. The slope of this negativity between 300 and 200 ms before EMG onset was significantly different from zero in the False Cognate condition (*t*(13) = −2.60, *p* = 0.022), but not in the Identity condition (*t*(13) = −1.45, *p* = 0.172). However, there was no significant difference between conditions on the slope of the negativity (*t*(13) = −1.25, *p* = 0.232). Finally, the peak-to-peak amplitude was larger in the False Cognate condition than in the Identity condition (*t*(13) = 2.47, *p* = 0.028). The topographies in Figure 3 indicate the fronto-central activity was well-centered at FCz.

In the Unrelated condition, the negativity peaked on average 160 ms (*SD* = 60 ms) before EMG onset, and the slope of this negativity between 300 and 200 ms before EMG onset was significantly different from zero (*t*(13) = 4.54, *p* < 0.001). Finally, as expected, the negativity was larger in the False Cognate condition than in the Unrelated condition (*t*(13) = 2.48, *p* = 0.027), but not larger in the Unrelated than in the Identity condition (*t*(13) < 1).

A left-lateralized component, at electrode F3, was found in the PWM task (Figure 3B). This negativity peaked on average 190 ms (*SD* = 37 ms) before EMG onset in the False Cognate condition and on average 232 ms (*SD* = 26 ms) before EMG onset in the Identity condition. There was an effect of condition on the latency of the peak (*t*(13) = 3.91, *p* = 0.002), as the negativity peaked later in the False Cognate condition than in the Identity condition. The slope of this negativity between 300 and 200 ms before EMG onset was significantly different from zero in the False Cognate condition (*t*(13) = −2.51, *p* = 0.026), but not in the Identity condition (*t*(13) = −0.29, *p* = 0.218). However, there was no significant difference between conditions on the slope of the negativity (*t*(13) = −1.57, *p* = 0.140). Finally, there was no significant difference between the peak-to-peak amplitude in the False Cognate condition and the Identity condition (*t*(13) = 1.00, *p* = 0.334).

In the Unrelated condition, there was no apparent negativity at electrode F3; the slope of the EEG waveform was not significantly different from zero between 300 and 200 ms before EMG onset (*t*(13) < 1). The topographies in Figure 3 indicate that the left frontal activity may be more anterior and inferior in the Identity condition compared to the False Cognate condition. We therefore also examined the activity recorded at F5, F7, AF3, and AF7 (see Figure S2) and found that the slope of the waveforms at these electrodes was not significantly different from zero in any of the conditions under analysis (all *t*s were between 0.28 and −1.09, except for F5 in the Identity condition: *t*(13) = −1.77, *p* = 0.099, and F7 in the Identity condition: *t*(13) = −1.88, *p* = 0.082).

## DISCUSSION

This study’s aim was to compare frontal cognitive control processes as engaged in linguistic versus non-linguistic decision-making in Spanish-English bilinguals using a PWM task involving false cognates and the arrow version of the Eriksen flanker task. Behavioral results showed that congruency effects were present in both the linguistic and non-linguistic tasks, but that the size of the congruency effects in the two tasks was not significantly correlated across participants. EEG results revealed a medial frontal component corresponding to the N-40 in the non-linguistic task. Its amplitude was modulated by congruency. A similar potential, although peaking earlier, around 150 ms before EMG onset, was found at the same electrode in the linguistic task and was modulated by linguistic congruency. Moreover, a left frontal potential was found in the linguistic task peaking around 200 ms before EMG onset and was sensitive to congruency. This component was absent in the non-linguistic task. These findings suggest a partial overlap between the control networks underlying the non-linguistic and linguistic tasks.

### Behavioral Results

Although the expected congruency effects were present in both the linguistic and non-linguistic tasks, the reaction times and the size of the congruency effects were different in the two tasks. The reaction times were shorter in the flanker task (between 394 and 466 ms on average) than in the PWM task (between 714 and 747 ms on average), but the congruency effect was larger in the flanker task (a 70 ms difference and 17% less accurate on average in the Incongruent compared to the Congruent condition) than in the PWM task (32 ms difference and no difference in accuracy on average in the Incongruent, i.e., False Cognate, compared to the Congruent, i.e., Identity, condition). The size of the congruency effect is on par with what was reported in the original study using the arrow version of the flanker task (74 ms in Stoffels & van der Molen, 1988), and the reaction times were in the same general order (between 350 and 450 ms). The size of the cross-linguistic interference effect is more variable in the literature varying between over 100 ms to less than 10 ms (van Heuven et al., 2008; Vanlangendonck et al., 2019; von Studnitz & Green, 2002); however, the tasks used in these studies were different than in ours and often used lexical decision to assess cross-linguistic interference rather than picture-word matching. In addition, interference resolution abilities have been shown to be modulated by language proficiency and dominance in bilinguals (Robinson Anthony & Blumenfeld, 2019), which may be another factor underlying the variability in the size of the cross-linguistic interference effect across studies. In a recent review, Calabria et al. (2018) note that how control is exercised is likely task-dependent and depends on bilingual profiles, calling for continued research into the exact manner in which bilingual control and executive function networks are linked in particular contexts.

Possible reasons why reaction times are longer overall in the PWM task could be linked to the fact that there are more possible response alternatives in this task overall (i.e., more than one possible name for a picture, and co-activation of linguistic representations that are related but not directly relevant to the experimental context), whereas there are only two possible responses in the flanker task. In addition, arrows are highly learned symbols and have very strong associations with their corresponding directions (Ridderinkhof et al., 2020). The associations between pictures and their names are generally not as high. Finally, it is possible that, as the central target arrow on the flanker task is spatially separated from its flankers, it was easier for participants to focus on the central target arrow and ignore flankers during flanker task performance than to focus on the picture and ignore the superimposed word during the PWM task.

Concerning the difference in the size of the congruency effect, one reason could be that in the flanker task, the alternative response is highly salient as the central arrow is flanked by four arrows going in the opposite direction in the incongruent trials. In addition, the association between these flanking arrows pointing in the direction opposite to the central arrow and the associated response is very strong, making the overcoming of this interference in response selection difficult. In contrast, in the PWM task, there is only one stimulus calling for the alternative response on the screen. In fluent readers, the superimposed text that cues the alternative response also yields an automatic reading response; however, this response is likely not as tightly associated with verbal output as the arrows in the flanker task are associated with right / left motor responses. Relatedly, bilinguals need to inhibit the other language constantly in everyday life and therefore likely have more practice in overcoming cross-linguistic interference associated with the superimposed text on the PWM task than non-linguistic interference as assessed in the flanker task.

### Medial Frontal Component

In the flanker task, we observed longer reaction times and lower accuracy rates in the Incongruent compared to the Congruent condition, replicating the well-established congruency effect in the arrow version of the Eriksen flanker task (Stoffels & van der Molen, 1988; for a review, see Ridderinkhof et al., 2020). In addition, our EEG results revealed a negative component peaking around 50 ms before EMG onset in the Incongruent condition. In the Congruent condition, there was no significant component peaking around the same latency, instead there was a later negativity peaking right around EMG onset. Previous reports have described a negative component peaking around 40 ms prior to EMG onset, at the same fronto-central recording site as in our study, in tasks requiring a choice between two possible responses (Carbonnell et al., 2004; Vidal et al., 2003, 2011). This component is thought to originate from the medial frontal cortex, including the SMAs (Vidal et al., 2003), and develops prior to the activation of the primary motor areas in tasks involving a choice to make between different possible manual responses (Carbonnell et al., 2004; Vidal et al., 2003). This component was also found to be modulated in amplitude by congruency in the Eriksen flanker task (Roger, 2009) and was found to be reduced in amplitude in situations when information about the response to be produced was available to the participant ahead of stimulus presentation (Carbonnell et al., 2004).

Our results are therefore largely in agreement with these findings, even if we did not find a negativity peaking at around the same latency in congruent trials. We note, however, that in contrast with the Incongruent condition, the slope of the waveform in the Congruent condition reported in Roger (2009) was also not different from zero between 90 and 60 ms pre-EMG onset, even though it was significantly different from zero in the later time window, between 60 and 30 ms pre-EMG onset. This is compatible with our observations even if the negativity peaked at around the same latency in congruent and incongruent trials in Roger (2009). Moreover, other studies investigating this component have reported an absence of negativity in the Congruent or easier condition, contrasting with the presence of a negativity peaking around 40 ms before EMG onset in the Incongruent or more difficult condition (Experiment 1, Roger, 2009; Carbonnell et al., 2004). Therefore, our results fall within the range of expected findings based on the literature.

In the linguistic task, we observed the expected cross-linguistic interference effect on the behavioral results. Performance was worse in the False Cognate condition than in the Unrelated and Identity conditions, as in other studies (e.g., van Heuven et al., 2008; Vanlangendonck et al., 2019; von Studnitz & Green, 2002). EEG results revealed a fronto-central negative component at the same recording site as in the non-linguistic task (FCz) peaking around 160 ms before EMG onset in the Unrelated condition. This component peaked around 30 ms earlier in the Identity condition, and 20 ms later in the False Cognate condition. The peak latency of ERP components has been reported to be modulated by factors such as task difficulty, age, and stimulus-onset asynchrony (e.g., Eddy & Holcomb, 2010; Kutas & Federmeier, 2011; Picton, 1992). The fact the negativity peaked later in the most difficult condition and earlier in the easiest condition is therefore not surprising. This is also in agreement with the results of Rodriguez-Fornells et al. (2006). This study found increased amplitude for the fronto-central negativity in the cross-linguistic phonological interference condition in both go and no-go trials, but also found a delayed peak latency for the same component in the no-go trials, requiring enhanced inhibitory control, compared to the go trials. Similarly, as in the non-linguistic task, the amplitude of this negativity was larger in the False Cognate condition (analogous to the Incongruent condition in the non-linguistic task) than in the Identity condition (analogous to the Congruent condition in the non-linguistic task). In addition, the amplitude of this component was larger in the False Cognate condition than in the Unrelated condition.

As mentioned in the Introduction, previous studies have found that bilinguals engage medial frontal regions typically associated with executive control, including the ACC and the pre-SMA, when faced with cross-linguistic interference (e.g., Rodriguez-Fornells et al., 2005; van Heuven et al., 2008). For example, van Heuven et al.’s (2008) neuroimaging findings suggested that pre-SMA / ACC areas showed greater activity for Dutch-English interlingual homographs than for control stimuli in a lexical decision task that included response-level conflict. However, we note that similar medial frontal activity peaking around 250 ms before vocal onset was also previously reported in picture naming, without manual responses, using the same techniques and source modeling as in the current study (Riès, Janssen, et al., 2013), and using magnetoencephalography (Salmelin et al., 1994), even though these studies did not specifically target bilinguals and did not manipulate cross-linguistic interference. That these activities peaked earlier than in the present study may be due to task difficulty as these studies used a simple picture naming task with no overlapping distractors. In addition, activity in the pre-SMA and SMA has also been reported preceding vocal onset in language production fMRI studies not targeting bilinguals (e.g., Alario et al., 2006; Tremblay & Gracco, 2010). Moreover, high frequency repetitive transcranial magnetic stimulation of the pre-SMA has been shown to interfere with the volitional selection of words and oral gestures (Tremblay & Gracco, 2009). As has been previously proposed (e.g., Tremblay and Gracco, 2009; Riès, Janssen, et al., 2013), our results suggest that the medial frontal cortex is active when a choice has to be made, whether it be linguistic or not, and that the amplitude of this activity is modulated by the difficulty of response selection, suggesting the brain mechanism underlying the medial frontal activity is shared across the linguistic and non-linguistic domains. In bilinguals, this medial frontal activity seems to be similarly engaged in linguistic and non-linguistic response selection, suggesting a functional and anatomical overlap between the two domains. However, we do not believe that the engagement of this medial frontal activity in language is unique to bilinguals, but instead is present across all speakers and sensitive to response selection difficulty across domains. The current data suggest that in bilinguals activity in this area correlated with interference resolution *across* languages in addition to the other response selection contexts already documented in monolinguals. The results of our study do not allow us to say whether or not this medial frontal activity is larger in bilinguals than monolinguals as we did not include a monolingual group.

### Left Anterior Frontal Component

A left anterior component was also observed at electrode F3 in the linguistic task. This component peaked later in the False Cognate condition than in the Identity condition (on average 190 ms before EMG onset in the False Cognate condition and 232 ms before EMG onset in the Identity condition). This effect of cross-linguistic interference on peak latency is similar to the one observed on the medial frontal component at FCz. Although there was no significant difference in amplitude between the False Cognate and Identity conditions, the slope of the negativity was significantly different from zero only in the False Cognate condition. Interestingly, this component was found at a more anterior site than in a previous picture naming study (at FC5 in Riès, Janssen, et al., 2013). In addition, the left anterior frontal activity in the present study peaked earlier than in Riès, Janssen, et al. (2013), where it peaked right around vocal onset, and was therefore interpreted as being associated with response preparation. The fact that the activity we report here peaked earlier and was sensitive to cross-linguistic interference suggests it is associated with process(es) preceding response preparation, such as response selection or cognitive control processes helping to resolve cross-linguistic interference. This could be consistent with van Heuven et al.’s (2008) finding of activation in anterior left inferior prefontal cortex linked to stimulus-based conflict resolution in cross-linguistic false cognates, even though the spatial resolution of EEG is limited even after Laplacian transformation and hence prevents us from making definitive claims regarding the source of the component we observed. Our results are also more generally in agreement with a role of the left PFC in resolving cross-linguistic interference, as previously proposed (Abutalebi & Green, 2007).

This left-lateralized component was absent in the non-linguistic task, suggesting the underlying brain activity may be specific to the linguistic task. We aimed to align our linguistic PWM and non-linguistic flanker tasks in terms of loci of conflict, with both stimulus-based and response-based conflict expected to be present in both tasks. However, it is possible that the stimulus-based conflict that arose in the linguistic task was more extensive. The longer reaction times in the linguistic task are in agreement with the fact that a wider array of representations was accessed during response selection in the linguistic task. In the non-linguistic task, there were only two possible stimulus dimensions that could receive activation, those corresponding to the left and right arrows. In the linguistic task, however, many different word representations likely received activation from the picture to be identified and from the overlapping distractor word, and the availability of multiple conceptual representations likely interfered with selection. For example, a picture of a foot with the word *PIE* overlaid is likely to activate the linguistic representations for *foot* in both English and Spanish, as well as the baked treat *pie* in English. In addition, adjacent phonological and semantic representations may be activated in both languages (e.g., Shook & Marian, 2013; van Heuven & Dijkstra, 2010).

A related explanation for the presence of the left anterior frontal component in the linguistic but not the non-linguistic task could be linked to the rostro-caudal organization of cognitive control functions in the frontal cortex (Badre & D’Esposito, 2009; Badre & Nee, 2018), with more abstract or higher order rules being encoded in the more anterior regions, and more concrete or lower order rules being encoded in more posterior regions such as the premotor cortices. Indeed, the PWM task engages linguistic word retrieval and phonological matching processes leading to selecting the response corresponding to a match or a non-match between the picture and the overlapping word. The flanker task instead engages more simple stimulus-to-response mapping where only two possible responses can be activated, selected, and executed, without any complex matching process between the stimulus and the response. We must, however, remain cautious when interpreting spatial localization differences in our study given the low spatial resolution of EEG, even if we did use Laplacian transformation. This result also suggests that additional cognitive control may be engaged in the linguistic task as compared to the non-linguistic task, therefore suggesting that the functional overlap between the cognitive control networks engaged in language and outside of language in bilinguals is only partial, at least as revealed by the tasks we used. This could be linked to inherent differences between linguistic and non-linguistic decision-making processes or to differences between the tasks used. Indeed, our results differ from those of Coderre et al. (2016), who contrasted a semantic categorization task to a flanker task and found overlap in the left inferior frontal gyrus in bilinguals. Our results also differ from those of De Baene et al. (2015), who found that highly similar brain regions, including the lateral and medial PFC, were engaged in linguistic and non-linguistic switching using closely matched linguistic and non-linguistic tasks. In this study, the linguistic task consisted in naming pictures in different languages as cued by a preceding symbol. Importantly, there were no overlapping written distractor words on the picture. In closer alignment to our results, a magnetoencephalography study that was not specifically targeting bilinguals found left superior frontal activity between 350 and 650 ms post-stimulus onset was found to be larger in the semantically related compared to the unrelated condition on a picture-word interference paradigm (Piai et al., 2014). This activity was interpreted as reflecting lexical competition resolution. The left anterior frontal activity we report here follows a similar time course even if we studied it time-locked to the response and not the stimulus. Given that we also used a picture-word interference paradigm, our left anterior frontal activity may reflect a similar competition resolution mechanism, which would be needed only in the linguistic and not in the non-linguistic task.

### Limitation of the Current Study

The relatively low number of participants included in the final analyses (*n* = 14) is lower than what is typically recommended in ERP studies examining potential cognitive consequences of bilingualism (Cespón & Carreiras, 2020). We, however, note that the number of participants needed may be dependent on the type of signal processing and analysis performed. Previous studies of response selection components using EEG and Laplacian transformation have shown reliable results using a similar or even smaller number of participants (*n* = 12 in Carbonnell et al., 2004; Riès, Janssen, et al., 2013; Vidal et al., 2003, 2011). Nevertheless, increasing the number of participants would be ideal in future studies and could clarify the results reported here (e.g., the nonsignificant difference in amplitude between conditions in the linguistic task for the left frontal component). Therefore, the results of the present study should be considered as preliminary given that Laplacian transformation has not been broadly used before in the context of research on cognitive underpinnings of bilingualism.

### Conclusions

To conclude, our study indicates that in bilinguals medial frontal activity preceding response execution is engaged in cross-linguistic and non-linguistic cognitive control associated with response selection at different time points, further supporting shared neural implementation but also suggesting shared computational principles between linguistic and non-linguistic domains at the level of response selection. An additional left anterior frontal component sensitive to cross-linguistic interference was also present in the linguistic but not in the non-linguistic task. Our results therefore suggest a partial functional overlap between linguistic and non-linguistic cognitive control processes as engaged in the tasks we used, and that linguistic conflict resolution may engage additional left anterior frontal control processes in parallel with more domain-general response selection processes in the medial frontal cortex. These findings align with the notion that when bilinguals resolve interference across languages, they engage a neural network that is in part domain-general. It has been argued that such an overlap between linguistic and non-linguistic networks is the basis for a mechanism that may drive changes in cognitive control associated with bilingualism. Specifically, the engagement of non-linguistic networks during bilingual processing may yield further use and strengthening of such networks (e.g., Bialystok & Craik, 2010).

## ACKNOWLEDGMENTS

This work was supported by a grant from the McNair Scholars Program awarded to Martha N. Mendoza; NIDCD grant 1R21DC016985 to Stephanie K. Ries; and NINDS grant 5R37NS021135 to Robert T. Knight. (The content is solely the responsibility of the authors and does not necessarily represent the official views of the National Institutes of Health.) The authors would like to thank the participants for their involvement in this study.

## FUNDING INFORMATION

Stephanie Ries, National Institute on Deafness and Other Communication Disorders (https://dx.doi.org/10.13039/100000055), Award ID: 1R21DC016985. Robert T. Knight, National Institute of Neurological Disorders and Stroke (https://dx.doi.org/10.13039/100000065), Award ID: 2R37NS21135.

## AUTHOR CONTRIBUTIONS

**Martha N. Mendoza**: Conceptualization: Equal; Data curation: Lead; Formal analysis: Supporting; Writing – review & editing: Equal. **Henrike K. Blumenfeld**: Investigation: Supporting; Writing – original draft: Equal; Writing – review & editing: Supporting. **Robert T. Knight**: Funding acquisition: Lead; Project administration: Supporting; Resources: Lead; Supervision: Supporting; Writing – original draft: Supporting; Writing – review & editing: Supporting. **Stephanie K. Ries**: Conceptualization: Lead; Formal analysis: Lead; Funding acquisition: Supporting; Investigation: Equal; Methodology: Lead; Project administration: Lead; Resources: Equal; Supervision: Lead; Validation: Equal; Visualization: Equal; Writing – original draft: Equal; Writing – review & editing: Equal.

## Supplementary Material

Supporting Figure 1

Supporting Figure 2

Supporting Information

## TECHNICAL TERMS

False cognates: Words that overlap in form but not meaning in different languages; also known as interlingual homophones and homographs.
Event-related potentials (ERPs): Voltage fluctuations recorded through electroencephalography at the scalp that are time-locked to an event.
Laplacian transformation: The mathematical process by which estimates of radial current flow at the scalp, or current source density, are derived from the scalp-recorded electroencephalogram.
