# Supplementary figures and images for: Investigating the Link Between Linguistic and Non-Linguistic Cognitive Control in Bilinguals Using Laplacian-Transformed Event Related Potentials
Source: Neurobiol Lang (Camb). 2021 Dec 23;2(4):605–27. doi: 10.1162/nol_a_00056 (PMC8886518; doi:10.1162/nol_a_00056)

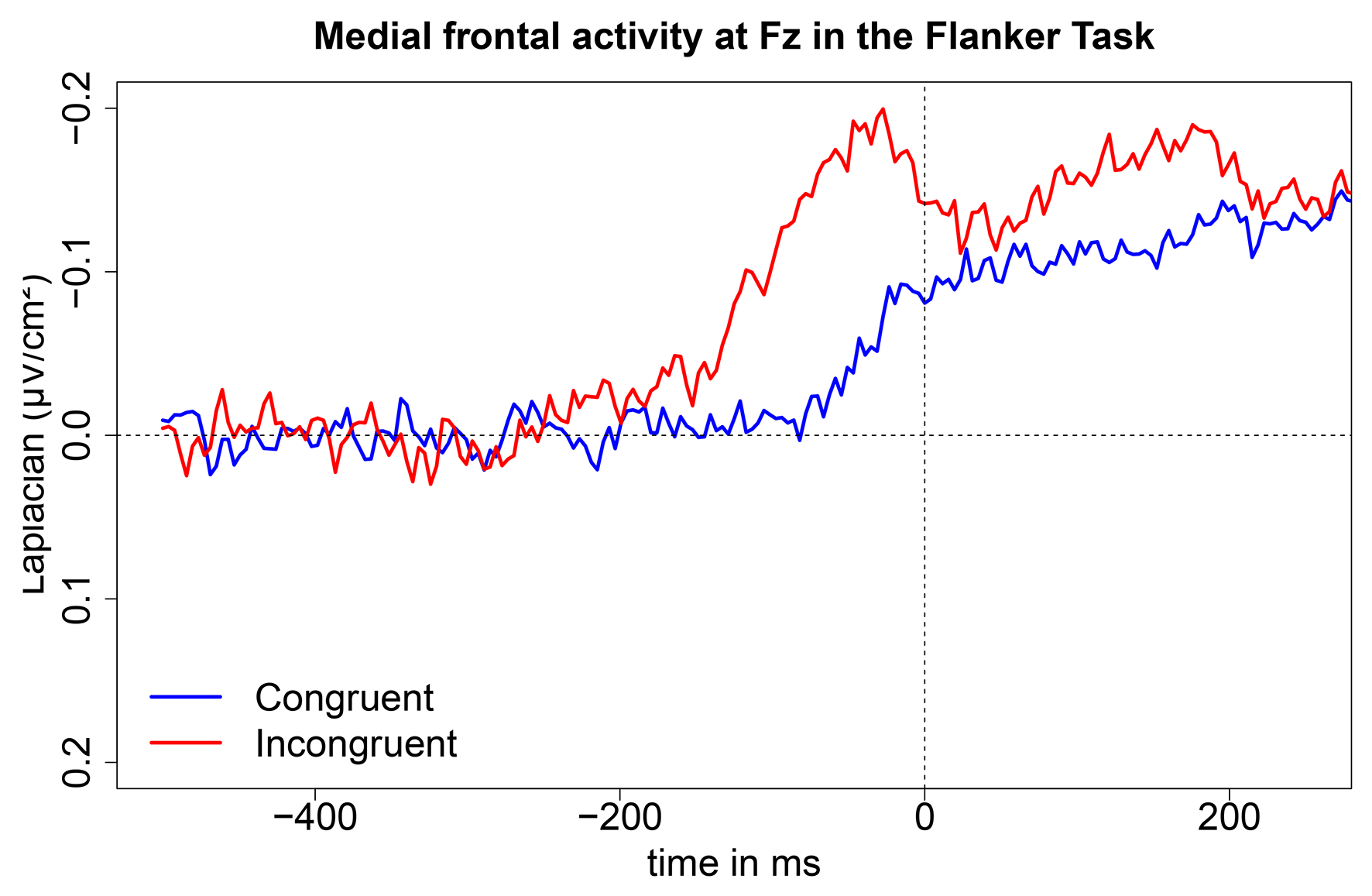

Supplement: Supporting Figure 1 [file figure_s1.png]

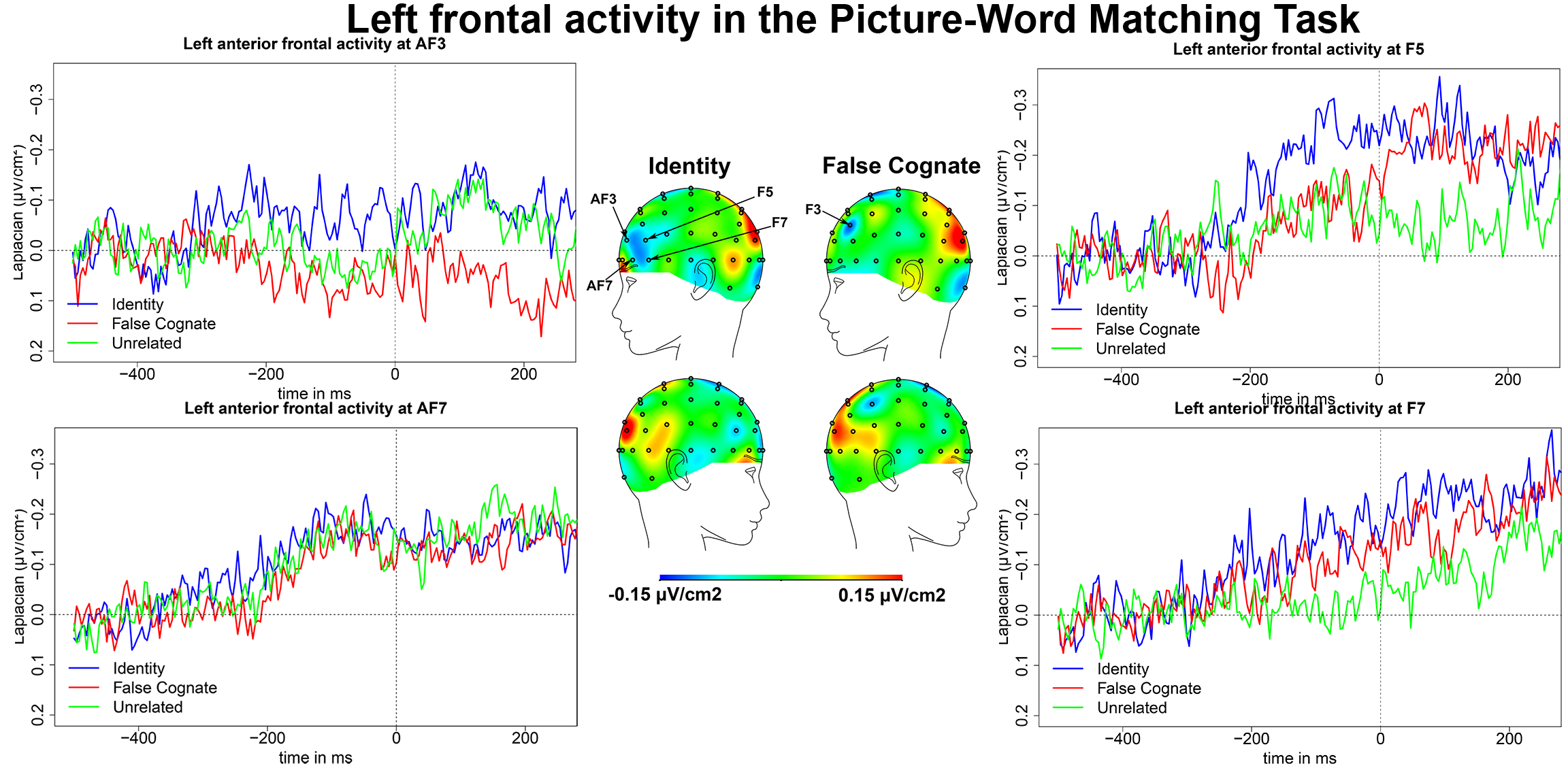

Supplement: Supporting Figure 2 [file figure_s2.png]
