# Supplementary material for: Investigating the Link Between Linguistic and Non-Linguistic Cognitive Control in Bilinguals Using Laplacian-Transformed Event Related Potentials
Source: Neurobiol Lang (Camb). 2021 Dec 23;2(4):605–27. doi: 10.1162/nol_a_00056 (PMC8886518; doi:10.1162/nol_a_00056)
Supplement: Supporting Information [file supplementary_information_nol_a_00056_mendoza.docx]

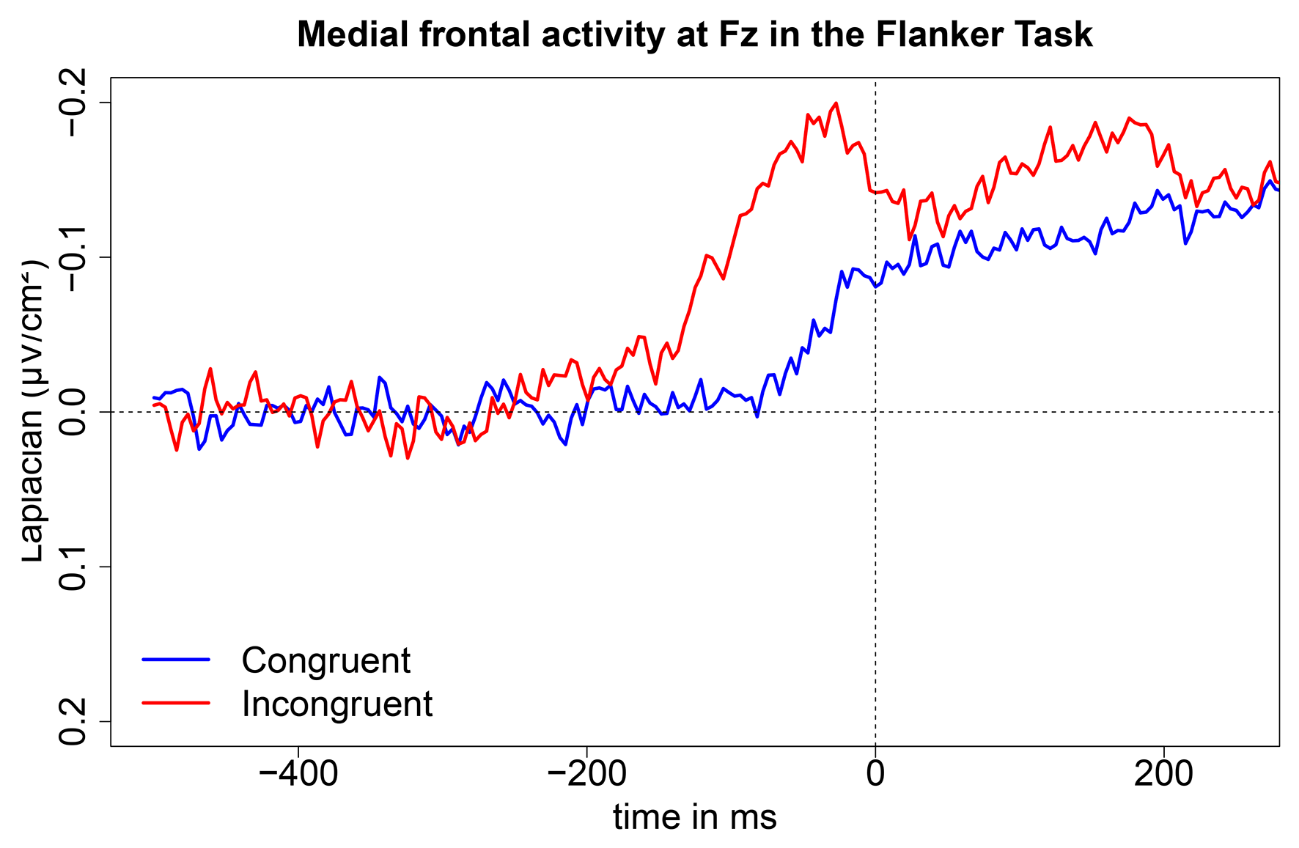


Figure S1: Medial frontal activity at Fz in the Flanker task in the congruent (blue) and incongruent (red) conditions time-locked to EMG onset (in ms).


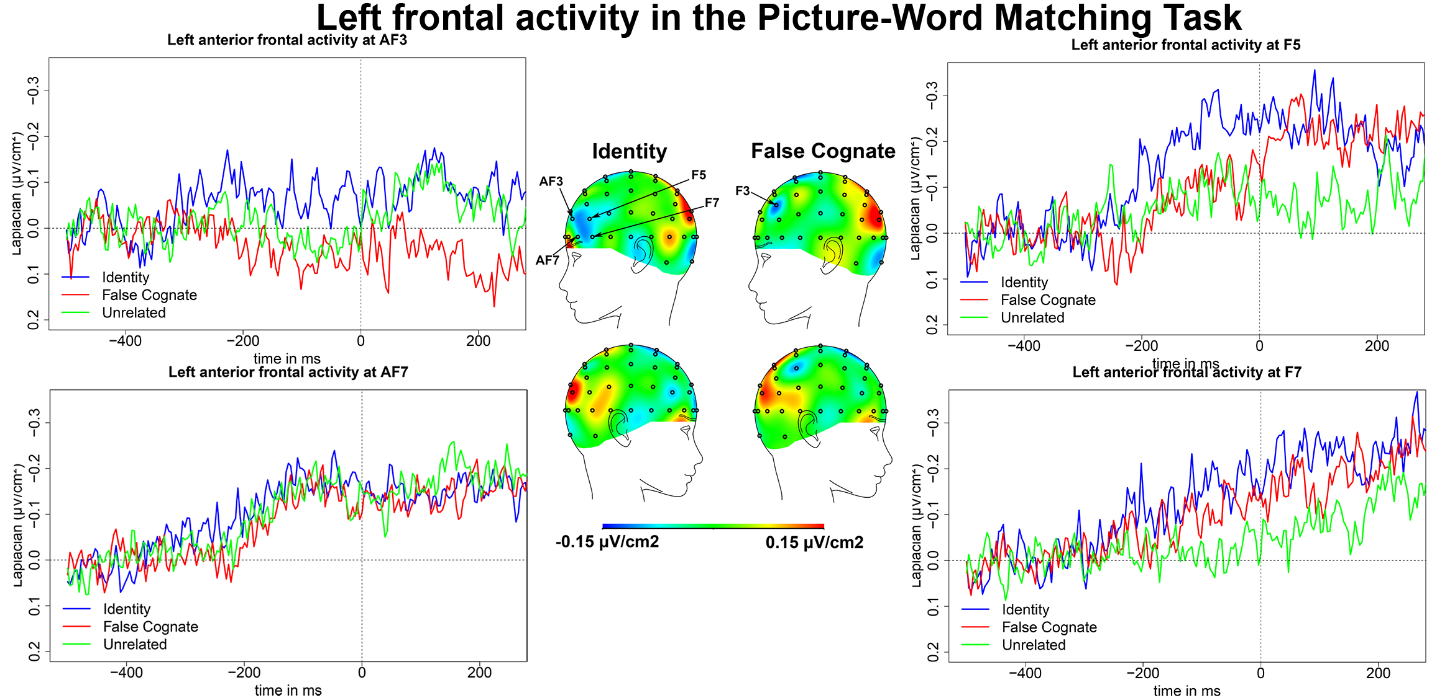


Figure S2: Left anterior frontal activity in the Picture Word Matching task at F5, F7, AF7, and AF3 in the Identity (blue), False Cognate (red), and Unrelated (green) conditions time-locked to EMG onset (in ms). In the middle, topographies of the left anterior frontal activity in the Identity and False Cognate conditions on a 50-ms time-window centered around the peak latency in the each condition.

**Appendix**: Stimuli - False cognate stimuli were identical or near-identical homographs of the Spanish translations of the English target picture name. The task was to decide if English target picture names matched overlaid text (either the target picture name in the identity condition, the false cognate distractor word, or the unrelated distractor word).

|  | Target picture name | Spanish translation of picture name | False-Cognate Distractor Word | Unrelated Distractor Word |
| --- | --- | --- | --- | --- |
| 1 | anchor | ancla | ankle | room |
| 2 | arm | brazo | brass | fly |
| 3 | balloon | globo | glove | anchor |
| 4 | bread | pan | pan | candle |
| 5 | candle | vela | veil | lollipop |
| 6 | card | tarjeta | target | milk |
| 7 | clothes | ropa | rope | wine |
| 8 | door | puerta | port | card |
| 9 | elbow | codo | code | door |
| 10 | envelope | sobre | sober | net |
| 11 | factory | fabrica | fabric | staples |
| 12 | feather | pluma | plum | factory |
| 13 | firefighter | bombera | bomber | clothes |
| 14 | flower | flor | floor | oven |
| 15 | fly | mosca | mosque | glove |
| 16 | folder | carpeta | carpet | flower |
| 17 | foot | pie | pie | bread |
| 18 | grass | pasto | past | table |
| 19 | lightbulb | foco | focus | envelope |
| 20 | lock | candado | candid | elbow |
| 21 | lollipop | paleta | palate | lock |
| 22 | milk | leche | leech | firefighter |
| 23 | net | red | red | soup |
| 24 | oven | horno | horn | lightbulb |
| 25 | pomegranate | granada | grenade | folder |
| 26 | pregnant | embarazada | embarrassed | grass |
| 27 | room | habitación | quart | pregnant |
| 28 | soup | sopa | soap | Sun |
| 29 | staples | grapas | grapes | foot |
| 30 | sun | sol | sole | feather |
| 31 | table | mesa | mess | pomegranate |
| 32 | wine | vino | vine | arm |
